# The Influence of Serum Prostate-Specific Antigen on the Accuracy of Magnetic Resonance Imaging Targeted Biopsy versus Saturation Biopsy in Patients with Previous Negative Biopsy

**DOI:** 10.1155/2017/7617148

**Published:** 2017-10-11

**Authors:** Chao-Hsiang Chang, Hung-Chieh Chiu, Wei-Ching Lin, Tzu-Lung Ho, Han Chang, Yi-Huei Chang, Chi-Ping Huang, Hsi-Chin Wu, Chi-Rei Yang, Po-Fan Hsieh

**Affiliations:** ^1^Department of Urology, China Medical University Hospital, Taichung, Taiwan; ^2^School of Medicine, China Medical University, Taichung, Taiwan; ^3^Department of Radiology, China Medical University Hospital, Taichung, Taiwan; ^4^Department of Pathology, School of Medicine, China Medical University, Taichung, Taiwan; ^5^Department of Urology, China Medical University Beigang Hospital, Beigang, Yunlin, Taiwan

## Abstract

**Objective:**

We compared the prostate cancer (PCa) detection rates of targeted biopsy (TB) and saturation biopsy (SB) in patients with previous negative biopsy and the accuracy of TB and SB stratified by different serum prostate-specific antigen (PSA) levels.

**Materials and Methods:**

Overall 185 patients were enrolled. In the magnetic resonance imaging (MRI) group, 65 men underwent TB and SB. In the control group, 120 men underwent SB alone. The primary outcome was the difference in PCa detection rate between the MRI group and control group. The secondary outcome was the difference in accuracy between TB and SB in detecting clinically significant PCa by stratifying the patients in the MRI group into those with PSA < 10 ng/ml and PSA ≥ 10 ng/ml.

**Results:**

The detection rates for overall and clinically significant PCa were higher in the MRI group than in the control group (46.2% versus 20.9% and 43.1% versus 16.7%, both *p* < 0.001). In the MRI group, the accuracy of TB was higher than SB (94.7% versus 84.2%, *p* = 0.001) for the patients with PSA ≥ 10 ng/mL.

**Conclusions:**

Combining TB and SB achieved the best cancer detection rate. The accuracy of TB was better than SB in the patients with serum PSA ≥ 10 ng/mL.

## 1. Introduction

According to European Association of Urology (EAU) guidelines on prostate cancer (PCa), the standard method of diagnosing PCa is ultrasound-guided transrectal (TRUS) or transperineal laterally directed biopsy of the prostate with 10–12 cores [1]. However, on average, less than 0.05% of prostatic tissue is sampled in each biopsy session, and more than 30% of cancers are located in the anterior horn, apex, or transitional zone where transrectal biopsy cores are usually missed [2, 3]. Therefore, a 12-core TRUS biopsy of the prostate carries a false negative rate of up to 20% [4]. In patients with a persistently elevated level of serum prostate-specific antigen (PSA) after an initially negative prostate biopsy, saturation biopsy (SB) with more than 20 cores of prostatic tissue performed in a systemic fashion has traditionally been suggested to improve the diagnostic accuracy [5–7]. However, by increasing the number of biopsy cores, SB may induce more pain and even transient erectile dysfunction [8]. The issue of the overdiagnosis of low-risk cancers caused by SB can also increase the complexity of treatment [9].

With significant advances in the techniques and interpretation of prostate multiparametric magnetic resonance imaging (mpMRI) in recent years, the role of MRI-targeted biopsy (TB) has been established in the repeated biopsy setting to improve the detection rate of clinically significant PCa [10, 11]. Nevertheless, around 10% of cancers are missed with TB alone [12, 13]. In order to achieve a maximal PCa detection rate, some reports have advocated the combination of TB and SB [14–16]. In this study, we investigated the overall and clinically significant PCa detection rates of TB and SB in patients with previous negative biopsy and compared the accuracy of TB and SB stratified by different serum PSA levels.

## 2. Materials and Methods

### 2.1. Study Population

From March 2012 to December 2014, 185 consecutive patients with prior negative biopsy, persistently elevated serum PSA level, and normal digital rectal examinations underwent repeated prostate biopsies in a tertiary referral center. After institutional review board approval, the patients' clinical characteristics and biopsy results were retrospectively recorded and analyzed. Two of the patients had atypical small acinar proliferation (ASAP) or high-grade prostatic intraepithelial neoplasia (HGPIN) on prior biopsy. MRI was arranged before repeated biopsies according to the physicians' clinical considerations, and the patients were divided into an MRI group (*n* = 65) and control group (*n* = 120). We used the Standards of Reporting for MRI-Targeted Biopsy Studies (START) to report the MRI and the biopsy results [17].

### 2.2. Magnetic Resonance Imaging and Analysis

MRI was performed on a 3-T MRI scanner (Signa HDxt, GE Healthcare, Milwaukee, WI) with an eight-channel high definition (HD) cardiac array coil. The scanning protocol included T2-weighted imaging (T2WI), diffusion-weighted imaging (DWI), and dynamic contrast enhanced (DCE) MRI. DWI was acquired with *b*-values of 0 and 1000 s/mm^2^, and an apparent diffusion coefficient (ADC) map was also generated.

Two radiologists (W. C. L. and T. L. H.) with 4 and 11 years of experience, respectively, in mpMRI reviewed the images (March 2012 to June 2014 by W. C. L., July 2014 to December 2014 by T. L. H.) and identified all suspicious cancerous lesions. Each lesion was assigned a score using the European Society of Urogenital Radiology (ESUR) Prostate Imaging-Reporting and Data System (PI-RADS) [18]. First, an individual score for each sequence (T2WI, DWI, and DCE) was given, and then a sum score was calculated. A lesion with a sum score of more than 10 was taken to indicate a suspicious lesion, and it was marked on a picture archiving and communication system workstation (Infinitt Healthcare, Phillipsburg, NJ).

### 2.3. Biopsy Protocol

In the MRI group, 65 men underwent prostate mpMRI. After interpreting the mpMRI findings and identifying the most suspicious lesions as the target lesions (maximum three target lesions per patient), a urologist (P. F. H.) performed cognitive registration TB, followed by SB, using a biplane TRUS probe (BK Medical, Transducer 8818). At least two cores were sampled from each target lesion, and at least 16 cores were sampled systemically from the peripheral zone and transition zone. In the control group, 120 men underwent transrectal SB alone by the same urologist with at least 16 cores sampled.

### 2.4. Histopathology

The biopsy specimens were interpreted by an experienced uropathologist (H. C.). We used the Epstein criteria to define clinically significant PCa as Gleason score (GS) ≥ 3 + 4, two or more cores positive for cancer, or cancer involving more than 50% of one core [19]. The maximal cancer core length was also recorded.

### 2.5. Statistical Analysis

We converted the overall ESUR PI-RADS score (3–15 points) to PI-RADS version 2 score (1–5 points) [20] by checking each individual score for further comparison with other studies. The primary outcome was the difference in detection rates for overall and clinically significant PCa between the MRI group and control group. The secondary outcome was the difference in accuracy between TB and SB in detecting clinically significant PCa by stratifying the patients as those with a serum PSA level < 10 ng/ml and those with a PSA level ≥ 10 ng/ml in the MRI group. The chi-square test and Student's *t*-test were used to analyze categorical and continuous variables between the MRI group and control group, respectively. The McNemar test was used to compare the performance of different biopsy methods in the MRI group. All clinical data analyses were performed using SPSS version 21 (IBM Corp, Armonk, NY, USA).

## 3. Results

Table 1 lists the characteristics of the study population. There were no significant differences in age, PSA level, and prostate volume between the MRI group and control group. Suspicious lesions with a PIRADS score ≥ 3 were identified in 43 patients (66.2%) in the MRI group.

The overall detection rates for PCa were higher in the MRI group than in the control group (46.2% versus 20.9%, *p* < 0.001). The detection rate for clinically significant PCa was also higher in the MRI group than in the control group (43.1% versus 16.7%, *p* < 0.001). In addition, the maximal cancer core length was longer in the MRI group than in the control group (2.6 mm versus 0.8 mm, *p* < 0.001) (Table 2).

In the MRI group, TB detected 28 cancers (43.1%), of which 25 were clinically significant, whereas SB detected 22 cancers (33.8%), of which 21 were clinically significant. The detection rates for overcall PCa and clinically significant PCa were comparable between TB and SB (43.1% versus 33.8%, *p* = 0.11, and 38.5% versus 32.3%, *p* = 0.34, resp.). There were three clinically significant cancers detected by SB but missed by TB, including two with GS 4 + 3 and one with GS 3 + 4. On the other hand, seven clinically significant cancers were detected by TB but missed by SB, including one with GS 5 + 4, one GS 4 + 5, one GS 4 + 4, two GS 4 + 3, and two GS 3 + 4 (Table 3).

Of the patients with a PSA level < 10 ng/mL, the accuracy of TB and SB was identical in detecting clinically significant PCa. However, of the patients with a PSA level ≥ 10 ng/mL, the accuracy of TB in detecting clinically significant PCa was higher than SB (94.7% versus 84.2%, *p* = 0.001, chi-square test) (Table 4).

## 4. Discussion

The incidence of infectious complications significantly increases with every prostate biopsy taken [21]. Therefore, every effort should be made to increase the cancer detection rate of prostate biopsy, especially in patients with previous negative biopsy and clinically suspected PCa. In this study, we demonstrated that the cancer detection rate was higher using a combination of TB and SB than SB alone in patients with previous negative biopsy. In addition, for the patients with PSA ≥ 10 ng/mL, the accuracy of TB was better than SB in detecting clinically significant PCa.

Previous studies have indicated the importance of combining TB and SB. Using radical prostatectomy specimens as the reference standard, Radtke et al. reported that a combination of TB and SB could detect 97% of significant PCa, which was significantly better than mpMRI (85%), TB (79%), and SB (88%) alone (*p* < 0.001 each) [22]. Hansen et al. prospectively evaluated the combination of transperineal TB and SB in patients with previously negative biopsy, and they found that, in patients with high probability MRI lesions (PIRADS 5), a combination of TB and SB could achieve the highest detection rates of PCa with GS ≧ 3 + 4 [15]. Pepe et al. reported that transperineal SB missed 9% of cancers, all of which were in the anterior zone, and that TB improved the accuracy in diagnosing significant anterior PCa [16]. Consistent with previous literature, we used an MRI group and a control group to show that the detection rate for clinically significant PCa was higher with a combination of TB and SB than with SB alone (43.1% versus 16.7%, *p* < 0.001). Combining TB and SB also achieved a higher detection rate of clinically significant PCa than SB alone in the MRI group (43.1% versus 32.3%, *p* = 0.02). Nevertheless, in the MRI group, TB and SB missed three and seven clinically significant cancers, respectively. Therefore, combining TB and SB should yield the highest cancer detection rate.

Li et al. reported the impact of serum PSA level on the detection rate of transrectal biopsy in the initial biopsy setting [23]. They found that, in men with PSA > 10 ng/mL, SB would not improve the cancer detection rate. In our MRI group, there was no significant difference in the detection rate of clinically significant cancer by TB and SB (38.5% versus 32.3%, *p* = 0.34). The accuracy between TB and SB was also comparable for the patients with PSA < 10 ng/mL (*p* = 1). However, for those with PSA ≥ 10 ng/mL, the accuracy of TB was higher than that of SB (94.7% versus 84.2%, *p* = 0.001) in detecting clinically significant PCa. Serum PSA level has been correlated with the aggressiveness of PCa [24], and PCa with PSA ≥ 10 ng/mL is categorized as intermediate or high risk, possibly implying a higher Gleason grade or more often clinically significant cancer compared to those with PSA < 10 ng/mL. In a prospective analysis using whole-mount section slides, Junker et al. demonstrated that tumor aggressiveness was correlated with PIRADS score [25]. In addition, the diagnostic accuracy of the PIRADS scoring system was better for those with high-grade PCa [18]. Therefore, the accuracy of MRI-guided TB may be better for patients with higher PSA or aggressive PCa. In other words, MRI is even more important in patients with a serum PSA level ≥ 10 ng/mL, and it should never be omitted in these patients. In addition, a combination of SB and TB is recommended to overcome the minor false negative rate of TB.

Pepe et al. reported that about 70% of clinically significant PCa in the anterior zone was diagnosed on repeat biopsy [26]. In addition, TB was shown to improve the detection rate, volume, and grade of anterior PCa compared with systematic biopsies [27]. In our series, we did not include the anterior zone in SB cores. Therefore, some clinically significant cancers would have been missed with a possible subsequent decrease in accuracy of SB. On the other hand, Cerantola et al. demonstrated that TB with cognitive MRI-US registration allowed for an accuracy of 82% in achieving the correct target, but that anterior tumors were less likely to be successfully targeted [28]. Due to the limited number of lesions in the anterior zone on MRI, we did not evaluate the role of TB in detecting tumors in this region. The issue of detecting anterior tumors by different biopsy methods should be evaluated in future studies.

In our series, TB was performed with cognitive registration. Theoretically TB with software registration is less operator-dependent and offers more objective results. In a prospective study, Wysock et al. demonstrated that although TB with software registration was more histologically informative than TB with cognitive registration, the detection rates for Gleason sum ≥ 7 were similar [29]. Another comparison between software and cognitive fusion did not show any advantage of one fusion method over the other [30]. The American Urological Association (AUA) and Society of Abdominal Radiology (SAR) consensus statements stated that in the absence of image fusion platforms, cognitive targeting remains a reasonable approach in skilled hands [31].

There are several limitations to this study. First, this is a retrospective study with a limited number of cases. Second, the definition of clinically significant cancer followed the Epstein criteria. However, it is debatable whether the Epstein criteria are feasible in the era of TB [32]. Third, we performed SB transrectally instead of transperineally, so it was difficult to sample prostatic tissue in the anterior zone or apex. We used biopsy results as the reference test, but both SB and TB could have false negative results [33]. Ideally, radical prostatectomy should be the best reference test; however it is not practical and may carry positive selection bias. Fourth, the operator performing SB was not blinded to the MRI reports, and the radiologist was not blinded to the clinical data. However, our data represent real clinical practice. Finally, we only included patients with previous negative biopsy, so the results cannot be applied to those undergoing an initial biopsy.

## 5. Conclusion

Our findings highlight the importance of combining TB and SB to achieve the best cancer detection rate for patients with previous negative biopsy. In addition, we found that the accuracy of TB was better than that of SB in patients with serum PSA ≥ 10 ng/mL. Further studies are needed to evaluate the impact of different biopsy strategies in patients with various PSA levels.

## Figures and Tables

**Table 1:** Patient characteristics.

	MRI group	Control group	*p* value
Number of men	65	120	
Age, years, median (IQR)	64 (7.5)	66 (14)	0.19
PSA level, ng/mL, median (IQR)	10.9 (7.5)	8.1 (5.7)	0.17
Prostate volume, mL, median (IQR)	48 (29)	53.5 (24.7)	0.09
PIRADS score, number (%)			
3	9 (13.8%)	Nil	
4	16 (24.6%)	Nil	
5	18 (29.2%)	Nil	

**Table 2:** Comparison of biopsy results between the MRI group and control group.

	MRI group (TB plus SB)	Control group (SB only)	*p* value
Total number of cores, median (IQR)	20 (4)	18 (3.5)	0.017
Number of cores with any cancer, mean ± SD	2.03 ± 3.33	0.74 ± 1.84	<0.001
Gleason score, number (%)			<0.001
≦3 + 3	6 (9.2%)	8 (6.7%)	
3 + 4	9 (13.8%)	8 (6.7%)	
4 + 3	10 (15.4%)	6 (5%)	
≧4 + 4	5 (7.7%)	3 (2.5%)	
Maximum cancer core length, mm, mean ± SD	2.6 ± 3.9 (TB)	0.8 ± 2.3	<0.001
Cancer detection, number (%)			<0.001
No cancer	35 (53.8%)	95 (79.2%)	
Clinically insignificant cancer	2 (3.1%)	5 (4.1%)	
Clinically significant cancer	28 (43.1%)	20 (16.7%)	

SB: saturation biopsy; TB: target biopsy.

**Table 3:** Comparison of TB and SB in the MRI group.

	SB		
	No cancer	Clinically insignificant cancer	Clinically significant cancer
No MRI target	22 (33.8%)	0	1 (1.5%)
TB			
No cancer	13 (20%)	0	1 (1.5%)
Clinically insignificant cancer	1 (1.5%)	1 (1.5%)	1 (1.5%)
Clinically significant cancer	7 (10.8%)	0	18 (27.7%)

Data are shown as number (percentage). SB: saturation biopsy; TB: target biopsy.

**Table 4:** Performance of TB and SB in the detection of clinically significant cancer with different PSA levels.

	PSA < 10 ng/mL			PSA ≥ 10 ng/mL		
	TB	SB	*p* value	TB	SB	*p* value
Sensitivity	88.9%	88.9%	1	89.5%	68.4%	0.03
NPV	94.7%	94.7%	1	90.5%	76%	<0.001
Accuracy	96.3%	96.3%	1	94.7%	84.2%	0.001

NPV: negative predictive value, SB: saturation biopsy, and TB: target biopsy.
